# A new look at IgE beyond allergies

**DOI:** 10.12688/f1000research.18186.1

**Published:** 2019-05-24

**Authors:** Andrea J. Luker, Joseph C. Lownik, Daniel H. Conrad, Rebecca K. Martin

**Affiliations:** 1 Department of Microbiology and Immunology, Virginia Commonwealth University, Richmond, VA, USA; 2 Center for Clinical and Translational Research, Virginia Commonwealth University, Richmond, VA, USA

**Keywords:** IgE, helminth, alpha-gal, auto-immunity, lupus, tumor, cancer

## Abstract

Immunoglobulin E (IgE), though constitutively present at low levels, is most commonly studied in atopic disease where it plays a vital role in mast cell degranulation and in initiating a T helper 2 (Th2) response. With the advent of better detection assays, however, researchers are discovering the importance of IgE in actively contributing to many disease states and pathologies. This review will discuss the latest findings in IgE beyond its role in allergies and recently discovered roles for IgE in its cell-bound form on FcεRI-expressing effector cells like monocytes and dendritic cells. In terms of parasites, we will discuss helminth-induced IgE that appears to protect the worms from immune recognition and a tick-borne illness that elicits an IgE response against red meat. Next, we describe recent findings of how auto-reactive IgE can contribute to the progression of lupus and induce organ damage. Finally, we summarize the emerging roles of IgE in tumor surveillance and antibody-dependent cytotoxicity. We additionally discuss recent or ongoing clinical trials that either target harmful IgE or use the unique characteristics of the isotype.

## Introduction

There are many hypotheses regarding the origin of the immunoglobulin E (IgE) response and its conservation throughout evolution. The more widely accepted theories include host protection against metazoan parasites, environmental toxins, and venoms. Although IgE-mediated degranulation is an effective means of initiating a protective immune response, the process can also cause significant damage to healthy tissue. IgE has long been viewed as the notorious Ig behind type I hypersensitivities and related diseases such as allergic rhinitis, anaphylaxis, and asthma. Classic IgE-mediated activation occurs through FcεRI on mast cells and basophils. Multivalent antigens cross-link the receptor-bound IgE to trigger degranulation and the release of pre-formed mediators. This pro-inflammatory milieu recruits immune cells and initiates a T helper 2 (Th2) immune response.

Today, millions of individuals suffer from allergies, asthma, and auto-immunity. This rapid rise in atopic disease may be explained by the “hygiene hypothesis” ^1^, which has been widely reviewed ^2– 5^. Briefly, this theory states that lower incidences of infection in developed countries lead to inappropriate immune activation by IgE against common environmental antigens and self-antigens. This hypothesis can be expanded to include the recent upsurge in food allergies, especially among young children ^6, 7^. Recent research has suggested that a healthy and diverse gut microbiota can prevent the development of IgE-mediated food sensitivities ^8, 9^.

Because asthma and allergies are a wide-scale problem across the globe, the majority of research into IgE revolves around these pathologies. Advances in analytical techniques as well as expanding scientific knowledge, however, have propelled a new wave of research into the role of IgE beyond the field of allergies. This review will span the latest findings on IgE in non-atopic pathological conditions. We will discuss a new take on “protective” IgE in helminth infection, revelations within a tick-borne disease, self-reactive IgE in auto-immunity, and a novel role for IgE in both preventing and treating cancer. Furthermore, we will discuss recent or ongoing clinical trials using IgE as a therapeutic tool in a variety of these scenarios.

## IgE in helminth infection: Whom is it protecting?

It is traditionally understood that antibodies are produced by B2 cells. These cells undergo isotype switching and affinity maturation to produce antibodies with very high specificity toward an antigen. The resulting high-affinity, antigen-specific antibody response is important within the adaptive arm of the immune system ^10^. In the mouse, however, there is a lesser-studied subset of B cell, the innate-like B1 cell, whose importance has begun to be revealed. Reports have described B1 cells as participants in resistance against intestinal bacteria, defense from viral pathogens, generating auto-immune responses, and responding to parasites ^11– 14^. In each case, the role of the B1 cell is to immediately respond to infection with the production of low-affinity IgM or IgG antibodies ^15^. Their B-cell receptor (BCR) repertoire is composed of vastly poly-specific receptors, meaning that B1 BCRs recognize a broad range of antigens ^15^. The resulting antibodies, however, are a much lower affinity than those produced by B2 cells.

Gastrointestinal helminths are widely known to elicit a robust antigen-specific IgE response, although the reasons why are unclear because mast cells do not appear to play a major role in helminth clearance. Recently, our group showed that helminth infection also induces the production of low-affinity, poly-specific IgE ^16^. Interestingly, this IgE is not derived from traditional B2 cells but instead is made by the innate-like B1 cells. This challenges the current understanding that B1 cells produce only IgM and IgG. Furthermore, Martin *et al*. revealed a competition between high-affinity B2 IgE and low-affinity B1 IgE to trigger or block mast cell degranulation, respectively ^16^. Although mast cell degranulation does not clear the infection alone, it can enhance the anti-helminth response. We hypothesized that helminth may have evolved to induce this low-affinity B1 IgE within the host to *prevent* mast cell degranulation as a protective means of immune evasion.

The translational relevance of murine B1 cells in the human system, however, remains controversial because they are not well defined. The current markers used to identify human B1 cells are similarly found on activated cells and memory populations ^17^. Human studies are also limited to circulating cells for collection and examination. For these reasons, it remains to be seen whether B1-derived IgE plays a clinical role in human disease.

## IgE and ticks

Scientists have recently discovered a bizarre occurrence in certain tick-bitten individuals within the southern US and beyond. These patients develop a delayed anaphylactic reaction against red meat, and the average onset is 3 to 6 hours after ingestion ^18^. The underlying cause is an IgE response against galactose-α-1,3-galactose (α-Gal), a carbohydrate moiety commonly found on glycoproteins. In particular, α-Gal is widely expressed within mammals but not within humans ^19^. The unique nature of the intense hypersensitivity reaction to α-Gal has remained a curiosity. Some hypothesize that certain ticks produce α-Gal in order to hide from the immune system of mammals, which are tolerized to see α-Gal as “self” ^19^. Humans, which are considered accidental hosts for ticks, however, will recognize the tick-expressed α-Gal as foreign and mount an immune response.

Additional questions remain surrounding the sensitization process following the tick bite as well as the delay in symptomology. The IgE responsible for red meat allergy is unique because of its specificity. Instead of recognizing a specific epitope on a specific protein, this IgE targets the α-Gal moiety that can be present on a variety of proteins and even lipids. This provides potential for “cross-reactivity” among all mammalian meats, including beef and pork.

The several-hour delay was originally thought to be caused by ineffective dendritic cell (DC) function. A recent study, however, showed that the presence of α-Gal on modified proteins actually increases the efficiency of antigen uptake ^20^. The authors further suggest that α-Gal may protect proteins from degradation, thereby slowing the process of major histocompatibility complex (MHC) presentation and antigen recognition ^20^. As mentioned before, α-Gal is also present on lipids. Fattier meats have been reported to elicit more severe reactions in patients with red meat allergy ^21^. One final theory attributes the late symptoms to the inherently slow metabolism of fatty lipids, resulting in the delayed release of α-Gal antigens.

## A hidden role for IgE in auto-immune disease

At a basic level, auto-immunity is characterized by an immune response directed against “self”. Auto-reactive B or T cells recognize proteins or DNA fragments normally found in healthy tissue, causing severe and chronic damage. We have come to understand that some of these disorders are characterized by high levels of circulating auto-reactive IgM and IgG ^22^. With the advent of better detection assays, however, it is now apparent that auto-reactive IgE is also present within these patients. In addition, researchers have recognized some self-antigens that also show cross-reactivity with exogenous or environmental allergens through molecular mimicry. Numerous reports have highlighted the elevated IgE seen in patients with atopic dermatitis ^23^, chronic urticaria ^24^, rheumatoid arthritis ^25^, or bullous pemphigoid ^26^. Indeed, there have been many reviews covering the decades of IgE studies in these diseases ^7, 27– 30^. The latest published research, however, has focused on systemic lupus erythematosus (SLE), the most common form of lupus.

### IgE and basophils

SLE is characterized by chronic inflammation due to the overproduction of auto-antibodies. These antibodies, often against nuclear factors like DNA, form immune complexes (ICs) that deposit in tissues and cause organ damage. Several studies have revealed a role for IgE in SLE, especially in terms of IgE-DNA ICs ^31– 34^. Work by Juan Rivera’s group has shown an important role for basophils in both murine models and SLE patients. The authors showed that auto-reactive IgE ICs activate basophils to produce interleukin-4 (IL-4) and contribute to an overall Th2 skewing ^31^. This inflammatory environment resulted in increased auto-antibody production by plasma cells and the development of severe lupus nephritis ^31^. The group went on to show that IgE deficiency delayed disease onset and prolonged survival ^35^. To authenticate the clinical relevance, they found increased basophil activation in SLE patients above healthy controls as well as the abnormal presence of basophils in the lymph node and spleen of two patients with SLE ^31, 35^. Furthermore, the authors showed a clear association of high levels of anti-dsDNA IgE in SLE patients with lupus nephritis and a positive association between auto-reactive IgE and clinical disease activity ^31, 36^. Overall, the authors hypothesize that IgE and basophils contribute to the disease pathology by *amplifying* the immune dysfunction.

### IgE and dendritic cells

Significant strides toward understanding the role of IgE in SLE were made when another group revealed a role for plasmacytoid DCs (pDCs). Traditionally, pDCs are a specialized DC subset that produce type I interferons (IFNs) in response to bacterial or viral pathogen-associated molecular patterns (PAMPs) ^37^. Henault *et al*. designed a novel anti-dsDNA IgE molecule, which they used in conjunction with free DNA to form ICs (DNA-IgE IC) ^34^. These DNA-IgE ICs bound to FcεRI on pDCs to trigger robust IFNα production through endosomal Toll-like receptor 9 (TLR9) activation. When co-cultured with B cells, pDCs activated by DNA-IgE IC caused a significant expansion of plasma cells. Furthermore, the authors showed close proximity between pDCs and B cells in kidney biopsies from patients with SLE. This suggests that the pDC-mediated B-cell proliferation seen *in vitro* may also be operant in SLE patients *in vivo*.

As a testament to the importance of these and many other findings, a small clinical trial (ClinicalTrials.gov identifier: NCT01716312) was conducted to test the effectiveness of treating SLE with the anti-IgE monoclonal antibody, omalizumab. The investigators hypothesized that neutralization of the circulating auto-reactive IgE would limit basophil and pDC activation, thereby dampening IFNα production. In a newly published report summarizing the results, the authors describe a maintained improvement in disease activity, as evaluated by using a patient scoring system (Systemic Lupus Erythematosus Disease Activity Index 2000, or SLEDAI 2K). Upon the conclusion of the 32-week omalizumab treatment period, patient scores began to worsen. The authors additionally analyzed type I IFN gene signatures and reported a trend toward improvement in patients receiving omalizumab. Although it did not reach significance within their small cohort, this attenuation effect was enhanced in those participants with high baseline scores. Additional studies will need to extend these results to include a larger cohort of participants. The results, however, are promising and lay an important framework for verifying the pathological relevance of IgE in SLE and other auto-immune diseases.

Another potential approach to reducing the effects of circulating IgE is through the use of soluble IgE receptors. The low-affinity IgE receptor, FcεRII or CD23, is well known to exist in both soluble and membrane-bound forms. CD23 is involved in a complex regulatory axis that controls the level of production of IgE ^38, 39^. The high-affinity IgE receptor, FcεRI, exists mainly as a tetramer (αβγ2), although some human cells express a trimeric form lacking the amplifying beta-chain (αγ2) ^40, 41^. In 1993, one group created a recombinant, soluble version of the human FcεRIα ectodomain (rsFcεRIα) ^42^. Using rsFcεRIα both *in vitro* and *in vivo*, the group was able to completely block mast cell and basophil activation ^42^. In 2011, another group reported their discovery of soluble FcεRI (sFcεRI) within human sera ^43^. The roughly 40-kDa soluble form described by Dehlink *et al*. consists solely of the IgE-binding alpha-chain ^43^. sFcεRI has been demonstrated to compete with membrane-bound FcεRI to bind IgE ^43, 44^. Indeed, the authors hypothesize that this competitive mechanism could attenuate IgE-mediated DC activation ^41^.

Recently, Moñino-Romero *et al*. sensitized mice with IgE in the presence of sFcεRI and observed a reduction in the severity of experimental anaphylaxis ^44^. Interestingly, sFcεRI was also effective in attenuating anaphylaxis when given post-sensitization ^44^. Within 24 hours of administration, sFcεRI caused a reduction in the level of IgE bound to the surface of circulating basophils. Although it was found to be as effective as omalizumab in blocking IgE/FcεRI interaction ^44^, much more research is needed to fully understand the physiological role of sFcεRI.

## IgE and neoplastic disease

Cancer is a disease of excessive cell proliferation. The malignant cells become invasive, disrupting organ architecture and leaching nutrients from healthy tissue. Because uncontrolled growth can lead to organ failure and death, early detection is vital for patient survival. The body’s defense against malignantly transformed cells relies mainly on the activity of CD8 ^+^ cytotoxic lymphocytes (CTLs). Tumors, however, can induce massive immune suppression to limit the activity of these effector T cells.

To prevent cancers from growing unchecked, there is great need to find new avenues to reverse the tolerance and induce an immune response within the tumor microenvironment. A popular antibody-based therapy consists of checkpoint inhibitors, which target T cell–deactivating molecules such as CTL-associated protein-4 (CTLA-4) and programmed death ligand protein-1 (PDL-1) in order to recover cytotoxic activity. Although many antibody drugs are on the market, they are all IgG-based. With five different antibody classes, it is possible that another isotype will become the next breakthrough in treating neoplastic disease.

Several epidemiological studies have compared IgE levels with incidences of cancer ^45– 47^. The overall conclusion suggests that allergic disease may grant some protection against certain types of malignancies. This has led to the emergence of research into AllergoOncology, and there are several reviews of the subject ^48– 50^. Elevated levels of IgE have been reported in patients with various types of cancer ^51– 53^. Although there has not been any conclusive evidence for what purpose this IgE may serve or what antigens it may be specific to, several groups have investigated the unique role of IgE in tumor protection and divulged mechanisms through a variety of effector cells.

### IgE as a memory response

In a direct investigation of the role of IgE in tumor development, one group explored adenocarcinoma growth in IgE-knockout (IgE-KO) mice ^54^. In un-immunized mice, the authors saw no difference in the tumor growth rate compared with that of wild-type (WT) mice. However, immunization with irradiated tumor cells 2 weeks prior to proper tumor challenge caused delayed tumor growth in the WT but not IgE-KO mice. Furthermore, mice with higher levels of IgE-secreting B cells (KN1) grew smaller tumors than WT mice and were completely protected in the immunization model. This IgE-protective effect was shown to rely on FcεRI and CD8 ^+^ cells ^54^.

### Using IgE to detect tumor antigen

In addition to the classically IgE-activated mast cells and basophils, other cell types express FcεRI or FcεRII (CD23) or both. In particular, DCs are vital to both immune surveillance and priming immune responses. DCs take up free antigen through phagocytosis or capture IgG ICs carrying bound antigen, a process known as receptor-mediated endocytosis ^55^. These exogenous antigens then can be routed through a specialized pathway for presentation on MHC class I. This process, known as cross-presentation, permits antigen-specific activation of CD8 ^+^ CTLs. A 2015 report described a novel mechanism by which DCs can capture low levels of *free* antigen on FcεRI-bound IgE ^56^. The authors were able to successfully show cross-presentation of soluble antigen to CD8 ^+^ cytotoxic T cells, resulting in proliferation and CTL activity. Furthermore, using antigen-specific IgE in a tumor vaccine model resulted in prolonged survival and generation of memory response upon re-challenge ^56^.

### Auto-reactive IgE as a protective force

Crawford *et al*. showed a very different role for IgE in preventing epithelial carcinogenesis ^57^. Following topical application of a carcinogen, the group discovered an unexpected accumulation of IgE within the skin. DNA damage within the epithelial cells triggered a unique mechanism whereby γδ lymphocytes induced draining lymph node (dLN) B cells to produce a robust repertoire of auto-reactive IgE. Using mice that lacked either IgE ( *IgH-7 ^−/−^*) or FcεRI (FcεRIα ^*−/−*^), they specifically showed that IgE signaling was playing a protective role, as the genetic-deficient mice developed more aggressive tumors ^57^. This gives important insight into a previously unexplored immune mechanism that prevents the progression to epithelial malignancy.

### Receptor-mediated cytotoxicity

In experiments using purified human peripheral blood mononuclear cells (PBMCs) transferred into nude mice, Karagiannis *et al*. showed that treatment with tumor antigen–specific IgE (MOv18) caused monocyte recruitment into the tumor site ^58^. The group also reported prolonged survival in treated mice, explained in part by an increase in antibody-dependent cell-mediated cytotoxicity (ADCC) that they observed *in vitro*. Interestingly, when cytotoxicity experiments were performed with non-specific IgE or PBMCs obtained from allergic patients, there was a significant decrease in activity ^58^. The same group later found that IgE can induce both monocyte-driven ADCC and antibody-dependent cell-mediated phagocytosis of ovarian tumor cells through FcεRI and CD23, respectively ^52^.

An ongoing phase I clinical trial is using this chimeric MOv18 IgE, specific for the cancer-associated folate receptor-alpha ^59^. Another group is working to optimize the process of cloning and mass-producing recombinant IgE for use in pre-clinical and clinical studies ^60^. Together, these experiments suggest that tumor antigen–specific IgE can play a powerful role in eliciting and maintaining an immune response at the tumor site.

## Conclusions and Future directions

Although IgE is normally the least abundant antibody found in circulation because of its short half-life, there are certain conditions that exhibit high levels of IgE. Typically, we associate this increased IgE titer with atopic diseases such as allergies and asthma. Furthermore, there is the additional nuance of discerning between antigen-specific and total IgE. This was particularly important in understanding the competition between antigen-specific B2 IgE and poly-clonal B1 IgE in helminth infection. This lesson can be applied to the many epidemiological studies of helminth infection in allergic patients. Whereas one study may find a positive correlation in IgE, another may not. Most likely, the difference in findings is due to the examination of total IgE rather than helminth-specific IgE. The additional translation between animal studies with helminth infection and human studies has been difficult, as experimental outcomes in human studies are minimal or self-described observations, and longevity studies are hard to find.

Extremely high levels of other self-specific isotypes, such as IgM and IgG, are characteristic of certain auto-immune diseases. It was only recently that advances in basic research have allowed us to unmask the presence of auto-reactive IgE in a variety of pathologies. The expanded use of omalizumab in these conditions is seeing positive results with few side effects and thus will likely result in a powerful adjuvant therapy for diseases like SLE.

Multiple groups have uncovered unique roles for IgE in detecting malignant cells, slowing tumor progression, and even maintaining a memory response. An interesting feature of allergies is the lifelong sensitivity that can continue for years even after removing exposure to the antigen. Although it is unclear exactly how this long-term hypersensitivity is maintained, the high-affinity memory response could be a beneficial avenue for surveying for tumor recurrence. Additionally, because IgE binds so tightly to its high-affinity receptor, FcεRI, cell-bound IgE could be retained within the tumor site for longer than other antibody classes. This could be a novel means of extending the activity of therapeutic drugs as well as a targeted means to elicit an immune response within the suppressive tumor microenvironment.
